# Pediatric Antiphospholipid Syndrome: from Pathogenesis to Clinical Management

**DOI:** 10.1007/s11926-020-00976-7

**Published:** 2021-01-28

**Authors:** Silvia Rosina, Cecilia Beatrice Chighizola, Angelo Ravelli, Rolando Cimaz

**Affiliations:** 1 Clinica Pediatrica e Reumatologia, IRCCS Istituto Giannina Gaslini, Genoa, Italy; 2 Experimental Laboratory of Immunological and Rheumatologic Researches, Immunology and Rheumatology Unit, San Luca Hospital, IRCCS Istituto Auxologico Italiano, Via Zucchi 18, Cusano Milanino, 20095 Milan, Italy; 3 University of Genoa, Genoa, Italy; 4 Sechenov First Moscow State Medical University, Moscow, Russian Federation; 5 Department of Clinical Sciences and Community Health, University of Milan, Milan, Italy; 6 RECAP_RD, University of Milan, Milan, Italy; 7 Pediatric Rheumatology Unit, ASST G. Pini & CTO, Milan, Italy

**Keywords:** Antiphospholipid syndrome, Antiphospholipid antibodies, Pediatric, Thrombosis, Non-criteria manifestations, Pathogenesis

## Abstract

**Purpose of Review:**

Elucidating the pathogenic mechanisms mediated by antiphospholipid antibodies (aPL) might exert important clinical implications in pediatric antiphospholipid syndrome (APS).

**Recent Findings:**

aPL are traditionally regarded as the main pathogenic players in APS, inducing thrombosis via the interaction with fluid-phase and cellular components of coagulation. Recent APS research has focused on the role of β2 glycoprotein I, which bridges innate immunity and coagulation. In pediatric populations, aPL should be screened in appropriate clinical settings, such as thrombosis, multiple-organ dysfunction, or concomitant systemic autoimmune diseases. Children positive for aPL tests often present non-thrombotic non-criteria manifestations or asymptomatic aPL positivity. In utero aPL exposure has been suggested to result in developmental disabilities, warranting long-term follow-up.

**Summary:**

The knowledge of the multifaceted nature of pediatric APS should be implemented to reduce the risk of underdiagnosing/undertreating this condition. Hopefully, recent pathogenic insights will open new windows of opportunity in the management of pediatric APS.

## Introduction

Antiphospholipid syndrome (APS) is an acquired, systemic autoimmune disorder characterized by arterial and/or venous thrombotic events and pregnancy morbidity with persistently positive antiphospholipid antibodies (aPL) [1]. Pediatric APS is defined as the syndrome presenting before the age of 18 years; however, some authors adopt as cut-offs 16 or 21 years [2, 3]. Currently, there are no universally accepted validated criteria for pediatric APS, and classification criteria for adult-onset APS are usually applied to pediatric populations. The updated set of criteria, formulated in 2006 in Sapporo, requires at least one clinical event (proven vascular thrombosis in arteries, veins, or small vessels, and/or pregnancy morbidity) and at least one persistently positive (at 12 weeks or beyond) aPL test [1]. The laboratory criteria may be met by a positive lupus anticoagulant (LA), anticardiolipin (aCL) IgG or IgM at medium or high titer (> 40 GPL/MPL or > 99th percentile), or anti-beta2glycoprotein I (anti-β2GPI) IgG or IgM above the 99th percentile [4]. As pregnancy occurs rarely in pediatric age [4], translating the adult classification criteria to children implies that APS can be formally diagnosed only in case of unprovoked, minimally provoked, or atypical thrombosis [5]. However, there is a great difference in the prevalence of concurrent pro-thrombotic risk factors between adult and pediatric populations: arterial hypertension, hyperlipidemia, obesity, atherosclerosis, and smoking are all rarely observed in younger subjects. Given that thrombosis is a multifactorial event, as outlined in the below-discussed two-hit hypothesis, such epidemiological observation might explain why thrombotic events occur rarely in pediatric age, and almost invariably in case of high-risk aPL profile. In children, non-thrombotic APS clinical manifestations, such as thrombocytopenia, hemolytic anemia, and neurological disorders, often precede overt thrombosis [2]. In addition, in order to prevent over-diagnosis of the syndrome, Sapporo criteria specifically exclude superficial vein thrombosis, which is a common condition in elderly patients, especially in case of varicose veins. However, superficial vein thrombosis is rarely observed in children, warranting a diagnostic work-up that should include aPL. The above-discussed distinctive features of adult versus pediatric populations explain why applying the Sapporo classification criteria for APS to children might result in missed or delayed diagnosis [6••]. Accordingly, the evidence-based recommendations for diagnosis and treatment of pediatric and neonatal APS recently published by the SHARE (Single Hub and Access point for pediatric Rheumatology in Europe) initiative clearly state that the updated Sapporo criteria are specific but not sensitive enough for the diagnosis of APS in children, underlying the need for the development of new criteria incorporating the whole range of aPL-associated manifestations [2, 6••, 7].

Pediatric APS can present at any age during childhood, most commonly between 9 and 14 years of age [8–13]. Differently from adults, where the male/female ratio has been estimated at 1:5, in children with APS, there is no gender predominance [4, 8]. APS can be defined as either primary (PAPS), when isolated, or secondary (SAPS), when it occurs in combination with another autoimmune condition. In pediatric populations, PAPS and SAPS tend to be similarly distributed [14••]. It has also been suggested that children may progress more often from PAPS to SAPS [4]. Even more frequently, aPL test positive in children without relevant clinical events, the so-called asymptomatic aPL carriers. Insights into the biological meaning of aPL tests and the pathogenic relevance of autoantibody subsets might lead to a more accurate stratification of the risk of future complications and to the optimization of the therapeutic approach.

### β2 Glycoprotein I: the Main Antigen Targeted by Antiphospholipid Antibodies

A cutting-edge frontier in APS research has now been focusing on the physiologic relevance of β2 glycoprotein I (β2GPI), the main antigen targeted by aPL. This molecule displays an evolutionary conserved structure, which suggests a relevant biological function despite the fact that β2GPI-deficient mice are apparently healthy. According to recent findings, β2GPI acts at the crossroad between the innate immune system and the coagulation cascade [15••]. Indeed, β2GPI can not only neutralize lipopolysaccharide (LPS), but also tune the activation of complement: on one hand, it enhances the degradation of C3 by factor I, on the other, it activates the lectin pathway via the interaction with the mannose-binding lectin, mediated by its high carbohydrate content. The complement lectin pathway can in turn promote coagulation by inducing coagulation factors [16]. Furthermore, β2GPI modulates coagulation also directly, exerting both procoagulant (inhibition of procoagulant protein C, displacement of anticoagulant Annexin A5, prevention of the formation of the thrombomodulin/thrombin complex and anticoagulant (prevention of platelet aggregation induced by ADP and von Willebrand factor [vWF], inhibition of thrombin, factor Xa, and tissue activator of plasminogen) mechanisms, with a net prevalent procoagulant effect [15••].

β2GPI is composed of five domains, with domain (D) 5 being aberrant because of an allosteric disulphide bond and a positively charged loop of lysine residues deputed to interaction with anionic phospholipids (PL). In the bloodstream, more than 90% of β2GPI adopts a circular conformation, with D1 interacting with D5. Upon binding to cardiolipin and LPS, or following changes in pH and oxidative state, β2GPI opens up to a J-shaped conformation where cryptic epitopes are exposed. This is extremely relevant: the main epitope in β2GPI resides in the N-terminal D1, and becomes available for antibody binding upon β2GPI conformational changes [17].

Since loss of tolerance towards β2GPI is unlikely due to its high blood concentration, several mechanisms have been proposed to explain anti-β2GPI autoantibody production. It is currently believed that post-translational modifications in β2GPI structure, such as in oxidation or glycosylation patterns, promote neoepitopes formation and in turn favor autoantibody production, as documented by the higher rate of altered β2GPI described in patients. Interestingly, in the non-oxidized form, β2GPI not only is not immunogenic, but also protects against cellular stress and ischemic damage. The presentation of β2GPI as antigen can occur even at endothelial level via class II MHC, and β2GPI-reactive CD4+ Th cells have been found to be more abundant in the atherosclerotic plaque than in the circulation [15••].

### Pathogenic Mechanisms of Antiphospholipid Syndrome

**Fig. 1:**
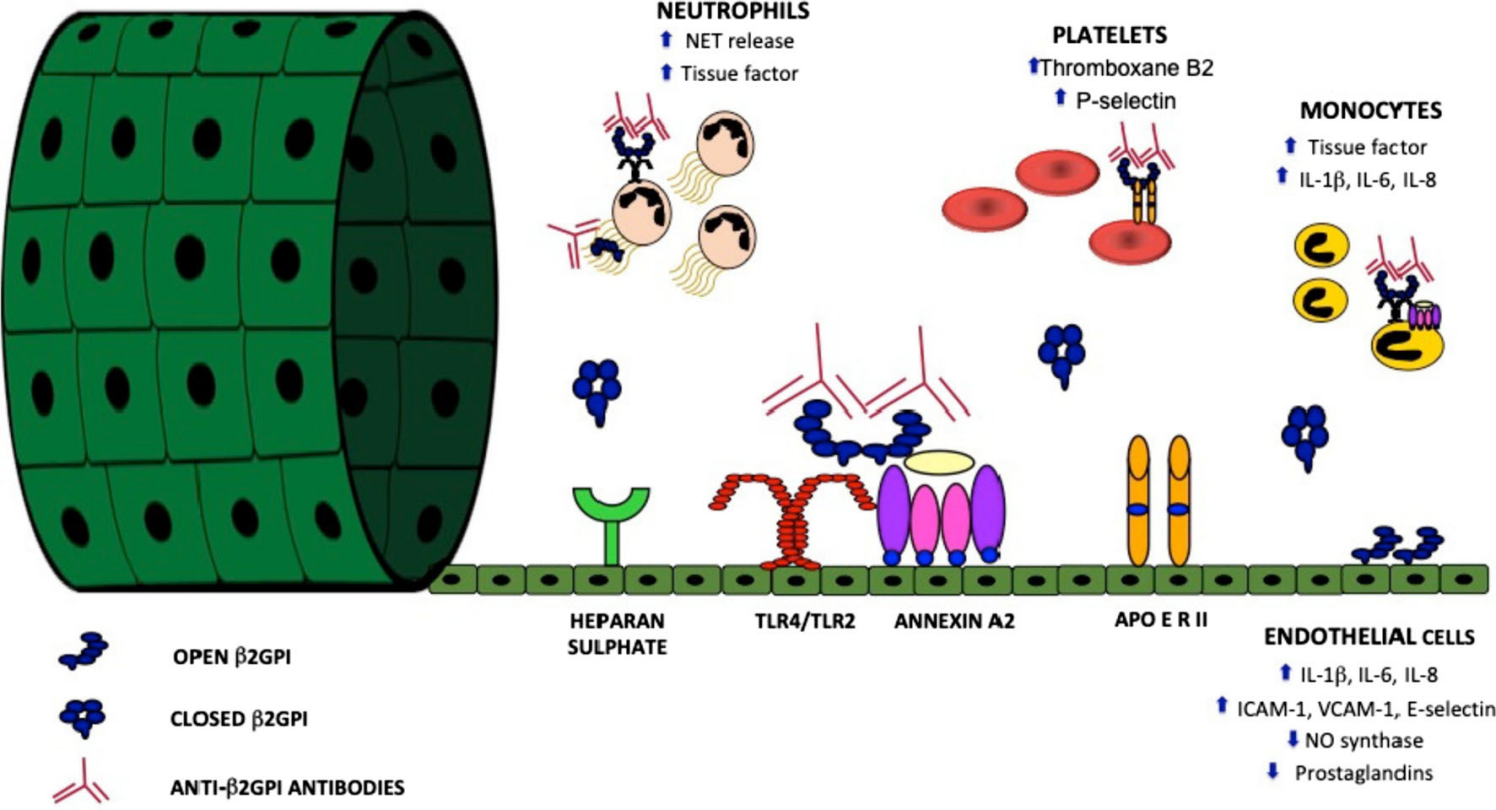
NET: neutrophil extracellular traps; IL: interleukin; ICAM: intercellular adhesion molecule; VCAM: vascular cell adhesion molecule; NO: nitric oxide; TLR: Toll-like receptor; Apo E R: apolipoprotein E receptor; β2GPI: β2 glycoprotein I

Anti-β2GPI antibodies exert their procoagulant potential by inducing a pro-inflammatory and pro-thrombotic phenotype in several cells involved in coagulation (Fig. 1). Indeed, aPL have been extensively documented in in vitro studies to engage β2GPI found on the cell surface of monocytes, platelets, endothelial cells, and neutrophils. The contribution of neutrophils to aPL-mediated hypercoagulatory state has been unraveled only recently: in vitro treatment with anti-β2GPI antibodies results in the release of higher levels of neutrophil extracellular traps (NET, which are networks of DNA complexed with histones and proteins) via Toll-like receptor (TLR) and adenosine A2A receptor activation [18•]. NET can directly activate the intrinsic coagulation pathway; synthetize tissue factor, the major initiator of clotting cascade; and provide an intravascular scaffold which facilitates the interaction between red cells, platelets, and coagulation factors. In APS patients, NET degradation is impaired, and neutrophils display a highly adhesive phenotype [18•]. In target cells, β2GPI might adhere to cell membrane via the binding of its D5 to anionic PL; in addition, several receptors have been proposed as potentially mediating anti-β2GPI/β2GPI interaction with target cells (Fig. 1). Most evidence favors Annexin A2 and TLR4, whose activation leads to the recruitment of intracellular mediators such as MyD88, NFκB, and p38MAPK. More recently, aPL have been shown to recruit in endothelial cells even the mammalian target of rapamycin, via the PI3K-Akt pathway [19]. aPL might also elicit vascular thrombi by interfering with fluid-phase coagulation factors (mainly prothrombin and thrombin), anticoagulant pathways (most notably, C protein and Annexin A5), and fibrinolysis (tissue activator of plasminogen). It is believed that such interaction is mediated by the cross-reactivity between β2GPI and conformational epitopes shared by several serin-proteases [20]. The complement system represents an emerging player in the pathogenesis of thrombotic APS, with β2GPI/anti-β2GPI antibody complexes resulting in the activation of the classical pathway. The relevance of complement has been documented in animal models, in reports of deposition of complement split products at the site of vascular thrombosis and in studies on serum complement levels in patients [21]. Despite in vitro data on aPL pathogenicity, treatment with Ig fractions is not sufficient to trigger vascular occlusion in experimental animals, requiring a “second hit” such as pre-treatment with LPS, mechanical or photochemical trauma. This observation parallels what happens in patients, who—despite the persistent positivity for circulating aPL—develop only occasional vascular events, often in concomitance of a hypertensive peak or an infection [20].

### Laboratory Detection of Antiphospholipid Antibodies

The above-discussed antigenic relevance of β2GPI and pathogenicity of anti-β2GPI antibodies is mirrored in the laboratory assays used in clinical practice to detect aPL. Indeed, anti-β2GPI antibody subpopulations are identified employing β2GPI as antigen, most commonly in immunoenzimatic or chemiluminescence assays; aCL tests usually detect antibodies reacting against cardiolipin complexed with β2GPI, and are thus referred to as “β2GPI-dependent aCL.” The latter assay allows identifying a broader antibody subpopulation, with a lower specificity for APS. When of G isotype and at high titers, anti-β2GPI antibodies induce the in vitro elongation of clotting time; this phenomenon, known as LA, is due to the interference of antibodies with PL function of essential cofactors in coagulation [22]. These observations account for the differential clinical relevance of aPL tests: LA is universally regarded as the most predictive test, whereas aCL positivity displays the lowest specificity but the highest sensitivity. In addition, patients with 2 or 3 positive tests are regarded at higher risk, and the hazard increases with the rising of antibody titers [23]. APS research is in a continuous quest of additional tests to further refine the process of risk stratification. In this regard, antiphosphatidylserine/prothrombin antibodies (aPS/PT) and antibodies against β2GPI-D1 (anti-D1) have emerged as the most promising assays. Anti-PS/PT are thought to mediate LA phenomenon in anti-β2GPI-negative subjects, and are significantly associated with both thrombotic and obstetric APS complications in a 2020 metanalysis [24••]. Because of their added diagnostic and prognostic value, anti-PS/PT were incorporated in the GAPSS score, an algorithm established to accurately estimate the magnitude of the thrombotic risk [25]. Anti-D1 antibodies provide a second-line test to be reserved to anti-β2GPI positive subjects, allowing to further characterize antibody pathogenic potential. Indeed, anti-D1 antibodies carry a prominent risk for vascular event, with an odds ratio of approximately 2 [19]. A multifaceted scenario emerges when evaluating anti-D1 antibodies and obstetric complications: anti-D1 reactivity predicts late complications, but not early abortion, potentially reflecting the placental resistance to ischemic damages in early gestation [26, 27]. Some authors have proposed aCL and anti-β2GPI IgA as more reliable APS biomarkers compared to IgM [28]. In particular, anti-β2GPI IgA have been found more promising than aCL IgA, especially in relation to thrombotic manifestations [29–32]. Conversely, many other studies have not described a positive association with APS manifestations [33–35] and adding aPL IgA to the current criterion panel does not increase odds ratios for thrombosis nor pregnancy morbidity [36]. Thus, no consensus has yet been reached on the inclusion of IgA aPL in the classification criteria for APS.

Several other autoantibodies not included in classification criteria (collectively named “non-criteria tests”) have been explored as potentially useful tools for diagnostic purposes especially in the so-called sero-negative APS, a term that refers to clinical manifestations of APS but negative conventional aPL tests: antibodies against phosphatidylethanolamine, phosphatidylserine, or phosphatidylinositol, to cite some. Despite some reports suggesting a significant association with APS clinical manifestations, the rate of isolated positivity of these tests is very low, thus not offering a real additive clinical significance [37•].

### In Utero Exposure to Antiphospholipid Antibodies

aPL-related pregnancy complications encompass abortions, both early and late, and premature birth occurring before 34 weeks, with a probability of overall pregnancy morbidity as high as 91% when both LA and anti-β2GPI IgG are positive [38••]. The above-cited aPL pro-thrombotic mechanisms can be shifted to obstetric APS, where intravascular thrombosis might lead to occlusion of uterine spiral arteries. Even neutrophils have been recently shown to be involved in the pathogenesis of aPL-associated pregnancy complications via the release of NET [39]. Similarly, the complement system, which plays a fundamental role in the physiologic human pregnancy, contributes to the pathogenesis of obstetric APS as suggested by in vivo models and studies of placental specimens from patients [21, 40]. However, important differences exist between thrombotic and obstetric APS, potentially due to the high β2GPI expression in the gravid uterus which facilitates aPL detrimental effects. Indeed, pregnancy complications can be reproduced in experimental animals via the passive transfer of aPL, not requiring a second hit [20], and aPL seem to be significantly associated with obstetric APS even when at low titers [38••]. Furthermore, histological examination of abortive specimens from APS women does not invariably document placental ischemic damage, even though areas of infarction are commonly reported in case of late losses [41]. Therefore, additional mechanisms have been advocated to mediate obstetric APS: aPL result in a decidual inflammatory and pro-apoptotic phenotype on the maternal side and in the disruption of the invasiveness, syncytialization, and proliferating potential of trophoblast on the fetal side [41].

Given this detrimental interaction not only with the maternal decidua but also with the developing fetus, it can be envisioned that prenatal exposure to aPL might lead to both short-term and long-term effects. Indeed, from the 14th gestational week on, the syncytiotrophoblast expresses the Fc receptor which mediates the passage across the chorionic villi of IgG [41]. Approximately 30% of maternal aPL can cross the placenta, potentially affecting fetal development. Due to the high tropism of aPL for the central nervous system, concerns have raised about the detrimental effects of these antibodies on the immature cerebral tissue, but no definitive conclusions on this debated issue can be drawn due to limited data. Learning disabilities have been described in 7 out of 33 children in two studies, while behavioral abnormalities were reported in 4 out of 134 babies included in the European registry of babies born to aPL-positive mothers [42•]. The AQUEOUS survey documented a lower rate of hospitalization and learning disabilities in 51 babies born after APS diagnosis compared to 48 born before [43].

At birth, it is an extremely rare finding to detect maternal IgG aPL in the cord blood and in neonatal circulation. In the aforementioned European registry, no episode of neonatal thrombosis was observed [44]. To date, 23 cases of neonatal thrombosis due to aPL have been described, a prototypic case of acquired autoimmune disease in which the pro-thrombotic state characteristic of the newborns, prominent in case of prematurity, might act as a “second hit.” Even less frequent is the occurrence of catastrophic APS (CAPS) in the perinatal life, with 3 described cases [42•].

Maternal IgG disappear from the offspring with time, being undetectable at 6–12 months. It has been shown that positivity for anti-β2GPI antibodies at 1 year of age does not depend on maternal aPL status, and that de novo antibody production peaks at 6 months of age and then decreases. It is believed that the ubiquitous presence of β2GPI in the environment might stimulate antibody production in children. Reassuringly, anti-β2GPI from 1-year-old children born to mothers with systemic autoimmune diseases preferentially target D4/5 of the molecules, which have been shown to be not pathogenic [42•, 45].

### Clinical Manifestations

A positive aPL profile can be found in children with a wide range of clinical manifestations, ranging from severe multi-organ life-threatening vascular events to asymptomatic positivity, as below reviewed.

#### Thrombotic Events

To date, the largest cohort of pediatric patients with full-blown APS is the International Pediatric APS (Ped-APS) Registry established by the European Antiphospholipid Antibodies Forum and Lupus Working Group of the Pediatric Rheumatology European Society. It includes 121 children from 14 countries, with a mean age at onset of 10.7 years [14••]. Most frequently, SAPS was diagnosed in children with systemic lupus erythematosus (SLE, 76.7%), followed by lupus-like disease (6.7%) and autoimmune thyroiditis (6.7%); APS was associated with malignancy in a single case. Patients with PAPS were younger and had more arterial thrombotic events, whereas SAPS manifested with a higher frequency of venous events associated with hematologic and skin manifestations [14••].

Venous thrombosis, in particular lower limb deep venous thrombosis (DVT), is the most common presenting manifestation of APS (Table 1). Among arterial thrombosis, ischemic stroke is the most frequently observed event, with a proportional incidence even higher than that reported in adults [14••]. Small-vessel thromboses are reported only in a minority of patients, and few children present both arterial and venous thrombosis [9, 14••]. Precipitating factors for thrombosis were identified in approximately one fourth of patients, including infectious events (43%), immobility (39%), surgery (11%), and trauma (7%); interestingly, 13% of patients had a family history of thrombosis [14••]. The recurrence rate of thrombosis in children has been estimated at 19%, higher than what reported for adult patients. Recurrent events tend to present in the same circulatory district as the presenting event [14••].

**Table 1:** Thrombotic events at presentation in two large case series of pediatric APS

Thrombotic event	Avcin [14••]	Ma [9]
	No. (%) of patients	No. (%) of events
Venous thrombosis	72 (60)	52 (77.6)
DVT in the lower extremities	49 (40)	25 (37.3)
Cerebral sinus vein thrombosis	8 (7)	3 (4.5)
Portal vein thrombosis	4 (3)	1 (1.5)
DVT in the upper extremities	3 (2)	–
Pulmonary embolism	–	17 (25.4)
Thrombus in the right atrium or inferior vena cava	–	4 (6.0)
Superficial vein thrombosis	2 (2)	–
Thrombosis in the left atrium	2 (2)	–
Jugular vein thrombosis	1 (1)	–
Inferior vena cava thrombosis	1 (1)	–
Renal vein thrombosis	1 (1)	2 (3.0)
Retinal vein thrombosis	1 (1)	–
Arterial thrombosis	39 (32)	15 (22.4)
Ischemic stroke	31 (26)	6 (9.0)
Peripheral arterial thrombosis	3 (2)	5 (7.5)
Retinal arterial thrombosis	2 (2)	–
Myocardial infarction	1 (1)	–
Renal artery thrombosis	1 (1)	–
Splenic infarction	1 (1)	1 (1.5)
Bone infarction	–	1 (1.5)
Testicular ischemia	–	1 (1.5)
Superior mesenteric artery thrombosis	–	1 (1.5)
Small-vessel thrombosis	7 (6)	–
Digital ischemia	4 (3)	–
Renal thrombotic microangiopathy	3 (2)	–
Mixed arterial and venous thrombosis	3 (2)	–
Ischemic stroke and portal vein thrombosis	1 (1)	–
Mesenteric artery and venous thrombosis	1 (1)	–
Renal artery and venous thrombosis	1 (1)	–

#### Non-criteria Manifestations

The clinical spectrum of APS has been increasingly recognized to extend beyond thrombotic and pregnancy-related events. Several additional clinical manifestations that do not reckon a purely pro-thrombotic etiology have been associated to aPL positivity but not included in the classification criteria due to a low specificity for the syndrome [1, 5, 46]. These “non-criteria” manifestations include hematologic, neurologic, dermatologic, cardiac, pulmonary, renal, and endocrine involvements, making APS a truly systemic autoimmune disorder (Table 2). From a clinical point of view, the most relevant complications include a plethora of neurologic manifestations, potentially due to the direct interaction of aPL with neurons, and aseptic valve vegetations, underpinned by a direct pathogenicity of autoantibodies. Thrombocytopenia, usually mild, might follow the neutralization by aPL of β2GPI binding to vWF, which results in enhanced platelet consumption [47].

**Table 2:** Non-criteria manifestations in two large case series of pediatric APS

Clinical manifestation		Avcin [14••]	Ma [9]
		No. (%) of patients	No. (%) of patients
Hematologic		46 (38)	–
	Thrombocytopenia	10 (8)	30 (52)
	Autoimmune hemolytic anemia	9 (7)	19 (33)
	Evans syndrome	15 (12)	–
	Leukopenia/lymphopenia	10 (8)	–
	Bleeding diathesis (e.g., LAHS)	2 (2)	–
Neurologic		19 (16)	–
	Migraine headache	8 (7)	–
	Chorea/athetosis	5 (4)	–
	Seizures/epilepsy	4 (3)	–
	Pseudotumor cerebri	1 (1)	–
	Mood disorder	1 (1)	–
	Transverse myelitis	–	–
	Cognitive impairment	–	–
	Ocular ischemia	–	–
	Stroke/TIA	–	–
Dermatologic		22 (18)	–
	Livedo reticularis	7 (6)	2 (3)
	Raynaud’s phenomenon	7 (6)	7 (12)
	Purpura fulminans	–	–
	Skin ulcers	4 (3)	1 (2)
	Pseudovasculitic lesions	3 (2)	4 (7)
	Chronic urticaria	1 (1)	–
Cardiac		–	–
	Valvular disease	–	3 (5)
	Myocardial infarction	–	–
Pulmonary		–	–
	Pulmonary hypertension	–	3 (5)
	Interstitial fibrosis	–	–
Renal		–	–
	Thrombotic microangiopathy	–	–
	aPL nephropathy	–	–
Endocrine		–	–
	Adrenal insufficiency	–	–
Articular		–	–
	Arthritis	–	12 (21)

*LAHS*, lupus anticoagulant hypoprothrombinemia syndrome; *TIA*, transient ischemic attack

#### Catastrophic APS

CAPS is a life-threatening presentation of APS, typically characterized by systemic inflammation and rapid (within 1 week) development of widespread microvascular thrombosis in multiple organ systems (3 or more), similar to other thrombotic microangiopathies [2, 5, 48, 49]. Preliminary classification criteria defining “definite” and “probable” CAPS were established and validated in adults and children [50, 51]. CAPS accounts for less than 1% of APS cases, being even less common among children. In the largest cohort of pediatric CAPS comprising 45 children, mean age at presentation was 11.5 years, 71.1% of patients were female, 68% had PAPS while 28.9% had concomitant SLE [52••]. Compared to adults, infections were more frequently identified as precipitating factor (60.9 versus 26.8%) and CAPS was more frequently the presenting manifestation (87 versus 45.2% in adults). Unfortunately, the *exitus* was observed in 26% of cases [52••].

#### Asymptomatic aPL Positivity

aCL and anti-β2GI can be found in 3–28% and 3–21% of otherwise healthy children, respectively [3, 53]. A positive LA can be detected in approximately 2% of apparently healthy children, usually discovered during pre-operative assessment of coagulation. These aPL are often transient and clinically insignificant, generally induced by infections or vaccinations [2].

#### aPL Positivity in Juvenile Idiopathic Arthritis and Systemic Lupus Erythematosus

aCL positivity has been detected in 30–53% of patients with juvenile idiopathic arthritis (JIA) [54, 55] but aPL-related thrombotic events are rarely seen [56, 57]. In a cohort of 28 JIA children, aCL emerged as the most frequently positive aPL test, also suggesting that the production of autoantibodies might follow an infectious trigger, and explaining the limited pro-thrombotic potential of aPL observed in JIA [58].

The prevalence of aPL positivity in SLE children exceeds 50%, a figure significantly higher than in adult lupus patients, with aCL being the most prevalent antibodies [4, 59–67]. Up to 21% of children initially diagnosed with PAPS progress to either SLE or lupus-like disease [8, 14••]. On the other hand, patients initially presenting with SLE may later develop APS. In a cohort of 57 children with SLE, 14% developed APS approximately 3 years after SLE diagnosis, most commonly presenting with arterial thrombosis (50%) [62]. aPL positivity provides the main risk factor for arterial and venous thrombosis in children with SLE. A cross-sectional cohort study of 979 pediatric SLE patients from CARRA registry showed an overall prevalence of arterial and venous thrombosis (independently of aPL status) of 2.5% and 3.6%, and the detection of any aPL significantly increased the overall thrombosis risk [54]. Another study followed up lupus children over 10 years, describing an annual thrombosis incidence of 5.4% for LA carriers and 2.2% for aCL carriers [4, 57]. Furthermore, aPL positivity in childhood SLE is an important predictor of irreversible organ damage, in particular renal and cerebral [61, 62, 67–70].

### Treatment

There is no difference in the acute treatment of thrombosis attributable to APS compared with other forms. Initial therapy in the acute setting consists of anticoagulation with either low-molecular-weight heparin (LMWH) or unfractionated heparin [4]. The SHARE recommendations suggest long-term anticoagulation therapy after a thrombotic event in case of persistent aPL positivity. It is important to highlight that direct oral anticoagulants should be avoided in APS patients, especially those with high-risk profile, as recommended by regulatory agencies due to the higher risk of recurrent events. Conversely, when aPL turn negative at further testing, long-term anticoagulation is not indicated [6••]. Recurrent thrombosis has been linked to insufficient anticoagulation in patients with APS [71]. In case of thrombotic recurrences despite oral anticoagulation, a higher target international normalized ratio (INR) (3.0–4.0 instead of 2.0–3.0) or alternative therapies (such as extended therapeutic dose of LMWH) should be considered [6••].

Conventional anticoagulation and/or antiplatelet treatments do not adequately control most of non-criteria manifestations, possibly because aPL-mediated inflammatory mechanisms have been implicated [72]. In general, the evidence for the treatment of non-criteria manifestations is limited to case series or case reports and is insufficient for general recommendations [72], especially in children. Thrombocytopenia in APS is usually moderate and rarely requires treatment. However, in patients with symptomatic thrombocytopenia, the same options available for immune thrombocytopenic purpura (steroids, intravenous immunoglobulins [IVIG], immunosuppressants, and splenectomy) should be considered [72]. First-line treatment for autoimmune hemolytic anemia in APS consists of high-dose corticosteroids, while traditional immunosuppressants, rituximab, or splenectomy have been used with inconsistent rate of therapeutic success as second-line treatments in refractory cases [72]. During the 14th International Congress on aPL, it was concluded that B cell inhibition may have a role in difficult-to-treat APS patients, possibly in those with hematologic manifestations. No standard treatment for non-criteria manifestations is available, and the role of anti-inflammatory drugs such as steroids, immunosuppressive agents or rituximab is yet to be defined [74]. Case reports showed successful treatment of aPL-associated chorea with hydroxychloroquine, mycophenolate, or IVIG, but prospective studies are needed to confirm their efficacy [72]. No specific treatment is usually needed for livedo reticularis, while in APS-related skin ulcerations, antiplatelets and/or anticoagulation are most commonly used [72]. Evidence-based recommendations for the management of heart valve disease in APS are lacking, but anticoagulation, antiplatelet, and immunosuppressive treatments may not reverse established valvular lesions or prevent their appearance [73, 75, 76].

Triple therapy (anticoagulation, corticosteroids, plasma exchange/IVIG) is considered the gold standard treatment for adult CAPS patients based on CAPS Registry [77]. The SHARE initiative recommends immediate combination treatment with anticoagulants, corticosteroids, and plasma exchange with or without IVIG in pediatric CAPS [6••]. In the CAPS Registry, none of the children who did not receive the combination treatment survived [52••]. Evidence regarding the use of biologics or immunosuppressive drugs is very limited in children with CAPS, while some evidence is available in adults. Based on 20 adult patients from CAPS Registry, rituximab may be beneficial for treatment of hematological and/or microthrombotic manifestations of CAPS [78]. Eculizumab, a complement inhibitor, has been quite successfully used in severe and/or recurrent CAPS [66, 67]. Since evidence in pediatric population is lacking, these medications should be prescribed with caution [6••].

Whenever instituting a primary prophylaxis, clinicians should take into account the aPL profile (e.g., single versus triple positivity) and other thrombotic or bleeding risk factors [5]. In adults, even though the only randomized controlled trial conducted in patients with persistently positive aPL (APLASA trial) failed to favor it in comparison to placebo in the prevention of thrombosis, low-dose aspirin (LDASA) was demonstrated to determine a significant decrease in the risk of first thrombotic event in aPL-positive patients in a large meta-analysis [79, 80]. However, because of bleeding risk during play and sports, long-term prophylaxis with LDASA is generally not recommended in asymptomatic children with aPL [2]. LMWH may be considered in high-risk situations such as immobilization or surgery [81]. In patients with childhood SLE and aPL, antiplatelets agents could be considered for primary thromboprophylaxis in addition to hydroxychloroquine [6••, 82, 83].

## Conclusions

The progressive unraveling of the proteiform nature of aPL subpopulations exerts strong implications in clinical practice, allowing clinicians to more and more accurately predict the hazard of future events. Such risk stratification should be pursued individually for each patient, a particularly relevant issue among pediatric aPL carriers [84]. The management of these cases might be tailored on the aPL profile: tight clinical control and primary prophylaxis in case of triple positivity, looser approach in case of isolated anti-D4/5 antibody positivity. It is imperative for the pediatric community to screen for aPL in the appropriate clinical settings, such as children with thrombosis or those presenting with multiple-organ dysfunction in the course of infection [4]. Pediatric rheumatologists should be well aware of the many patterns of APS presentations, so to early detect the whole range of potential aPL-related complications and institute a prompt management. Hopefully, the many non-criteria APS manifestations will be soon incorporated in a specific set of classification criteria for pediatric APS [6••, 85]. Similarly, to reduce the morbidity burden conveyed by aPL positivity, it is crucial to screen for aPL in pediatric lupus patients [6••]. Definitive conclusions on long-term deleterious effect of aPL exposure in utero cannot be yet drawn, also because in these instances prematurity should be adequately accounted for. Nevertheless, it is advisable to follow up children born to aPL-positive mothers to early identify developmental disabilities.

As a whole, the knowledge of the multifaceted nature of pediatric APS should be implemented to further reduce the risk of underdiagnosing or undertreating this condition. It is desirable that the recent insights into APS pathogenesis, in particular the elucidation of the physiologic role of β2GPI and the identification of novel cellular pathogenic players, will soon allow opening new windows of opportunity in the management of pediatric APS.
